# Quantifying Trends in Disease Impact to Produce a Consistent and Reproducible Definition of an Emerging Infectious Disease

**DOI:** 10.1371/journal.pone.0069951

**Published:** 2013-08-14

**Authors:** Sebastian Funk, Tiffany L. Bogich, Kate E. Jones, A. Marm Kilpatrick, Peter Daszak

**Affiliations:** 1 Institute of Zoology, Zoological Society of London, London, United Kingdom; 2 Department of Ecology and Evolutionary Biology, Princeton University, Princeton, New Jersey, United States of America; 3 London School of Hygiene & Tropical Medicine, London, United Kingdom; 4 EcoHealth Alliance, New York, New York, United States of America; 5 Fogarty International Center, National Institutes of Health, Bethesda, Maryland, United States of America; 6 Department of Genetics, Evolution and Environment, University College London, London, United Kingdom; 7 Department of Ecology and Evolutionary Biology, University of California Santa Cruz, Santa Cruz, California, United States of America; National Institutes of Health, United States of America

## Abstract

The proper allocation of public health resources for research and control requires quantification of both a disease's current burden and the trend in its impact. Infectious diseases that have been labeled as “emerging infectious diseases” (EIDs) have received heightened scientific and public attention and resources. However, the label ‘emerging’ is rarely backed by quantitative analysis and is often used subjectively. This can lead to over-allocation of resources to diseases that are incorrectly labelled “emerging,” and insufficient allocation of resources to diseases for which evidence of an increasing or high sustained impact is strong. We suggest a simple quantitative approach, segmented regression, to characterize the trends and emergence of diseases. Segmented regression identifies one or more trends in a time series and determines the most statistically parsimonious split(s) (or joinpoints) in the time series. These joinpoints in the time series indicate time points when a change in trend occurred and may identify periods in which drivers of disease impact change. We illustrate the method by analyzing temporal patterns in incidence data for twelve diseases. This approach provides a way to classify a disease as currently emerging, re-emerging, receding, or stable based on temporal trends, as well as to pinpoint the time when the change in these trends happened. We argue that quantitative approaches to defining emergence based on the trend in impact of a disease can, with appropriate context, be used to prioritize resources for research and control. Implementing this more rigorous definition of an EID will require buy-in and enforcement from scientists, policy makers, peer reviewers and journal editors, but has the potential to improve resource allocation for global health.

## Introduction

Infectious diseases are a significant threat to global health in the 21^st^ Century, causing significant morbidity and mortality, and economic loss [1]. However, the allocation of funding to research and control diseases is often based on subjective assessment, rather than rigorous estimates of the total current and anticipated disease burden, leading to large mismatches between impact and allocation [2], [3]. In addition, some diseases are labeled as “emerging” following an initial outbreak, but which cause relatively little public health impact subsequently, often garner significant attention and resources (e.g. hantavirus pulmonary syndrome in the USA; see below). Emerging diseases are generally considered those that have recently increased in impact, moved into new geographic regions, moved into human hosts for the first time, changed their clinical presentation with more severe symptoms, or are caused by newly evolved pathogens [4], [5], [6], [7], [8], [9], [10], [11], [12], [13]. These “emerging infectious diseases” or “EIDs” have become the focus of national [14], regional and global [15], [16], [17], [18], [19] control programs.

Despite heightened interest in EIDs and frequent use of the term “emerging” to draw attention to a disease, its use is subjective, inconsistent and incomplete and rarely is based on a reproducible quantitative analysis of the trend in impact. Developing a consistent and reproducible definition to EIDs is not simply a semantic issue because proper designation of a disease as “emerging” implies an increased impact in the future and is part of a valid utilitarian approach to determine the importance of different diseases and to the allocation of scarce public health resources [20]. An accurate and quantitative description of the trend in a disease's impact would thus allow public health managers to better define which disease are highest priority, and also to determine when an EID becomes endemic, or recedes in impact, so that resources could be allocated to other public health threats.

A rigorous analysis of trends in disease impact must overcome several challenges. First, authors usually do not put temporal bounds on emergence so that diseases for which incidence increased in a prior period, but is now diminishing, are still considered ‘emerging’. Although an influential review from the Institute of Medicine suggested a timescale of 20 years [8] over which to examine patterns of incidence, this is an arbitrary length, and regardless, defined time limits have rarely been used. Second, most previous studies do not quantify geographic variation in the change in incidence or impact. The key point is that to quantify the trend in the impact of a disease, we must define the population in which a disease is proposed to be emerging and the time period over which emergence has or has not occured.

In this paper, we illustrate a simple, reproducible and quantitative method to add objectivity to previous definitions of emergence [4], [5], [6], [7], [8], [9], [10], [11], [12], [13] based on a statistical analysis that can be applied to time series, including incidence data, or, preferably, measures of disease impact such as Disability Adjusted Life Years (DALYs). Our goal is to highlight the value of considering the trend in a disease's impact and by doing so add a measure of rigor to the term “emerging” which will restore it as an important and useful descriptor of a growing set of infectious diseases. In addition, several recent studies have aimed to uncover the trends and factors that cause the emergence of diseases [4], [21], [22] and these studies require identification of the point in time that a disease begins to emerge within a region and population. The analyses we describe provide a way to identify this point in time and will thus provide a way to better analyze the process of disease emergence.

## Methods

To develop a quantitative definition of disease emergence, trends in incidence (the number of cases a disease produces in a defined population within a given time period) or public health impact (which can be measured in a number of ways, e.g. mortality, morbidity, or DALYs) must be examined over a given time period and in a defined population. The simplest method for quantifying trends in disease incidence or impact is a linear regression with time as the predictor. This produces an increasing (emerging) or decreasing (recending) slope, or no significant change (stable) in incidence in the population over the time period examined. For diseases that have undergone changes in trends, an additional tool for examining time series, segmented linear regression, can be used. The model can be written as
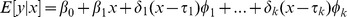
Where y are the incidence data, x is time, *β_0_* is the intercept, *β_1_* is the initial slope, the *τ_k_* are the unknown points of inflection (or joinpoints), *δ_k_* are the changes in slope after the inflection point, and the *φ_k_* are 1 if *(x-τ_k_)>0*, and 0 otherwise to ensure continuity at joinpoints. We determined the best fitting number and location of joinpoints by exhaustively exploring each year in the time series as a joinpoint. We tested whether a model with 1 or more joinpoints fit better than a null model that had fewer joinpoints (beginning with zero). We compared models using a permutation method in which the residuals of the null model (for 0 joinpoints the null model is a simple linear regression) are repeatedly permuted (randomly reordered) and added to the predicted values from the null model to generate thousands of permuted data sets [23]. The alternative model with additional joinpoints is then fit to the permuted data sets and the distribution of a goodness-of-fit measure (in this case an F-statistic or a simpler monotonic transformation of the F-statistic) is obtained. The alternative model with more joinpoints is favored if the improvement in goodness of fit against the null model obtained by fitting to the original data is highly unlikely; i.e. it is in the upper 5% percentile of all the improvements in goodness of fit on the permuted datasets. The method is conservative in the sense that in a situation where the data are not informative, the simpler model is chosen. This approach to select the number of joinpoints was developed to detect changes in trends in cancer rates [23].

To illustrate this approach we analyzed yearly incidence data for twelve diseases from the GIDEON database [24] from 1961 until 2010. GIDEON collates referenced data for 349 infectious diseases worldwide. Although GIDEON data contain some inaccuracies, they provide useful illustrations of the patterns that arise most frequently with case data. We performed segmented regression using a maximum of 2 joinpoints, a minimum of 5 years between joinpoints (both of which are arbitrary), 1000 permutations and an overall significance threshold of 0.05 with Bonferroni correction. We used an identity link function and assumed the data were Poisson distributed, and classified a disease as emerging or receding if, over the period between joinpoints, the slope was significantly greater or less than 0, respectively, with a significance threshold 0.05 using a z-test. The method was implemented in C++, and fitting was done using Levenberg-Marquardt nonlinear least squares. An independent GUI-based implementation of the method is available [25], and a similar method (based on iterative fitting) has been implemented in the open-access *segmented* package in R [26]. Recent work suggests that comparing among models with different joinpoints is best done either with the permutation method above, or with Bayesian Information Criterion, both of which are more conservative than Akaike's Information Criterion which often leads to overfitting [27].

The best fitting joinpoints (points of inflection) are estimates of the beginning of an emergence or receding event. Additionally, disease “re-emergence” can be rigorously defined using this approach. For a disease to be re-emerging, the incidence or impact of a disease must be shown to increase initially (the first emergence event), then either stabilize or recede, and then increase again (the re-emergence). These patterns are illustrated with actual incidence data below.

## Results

**Figure 1:**
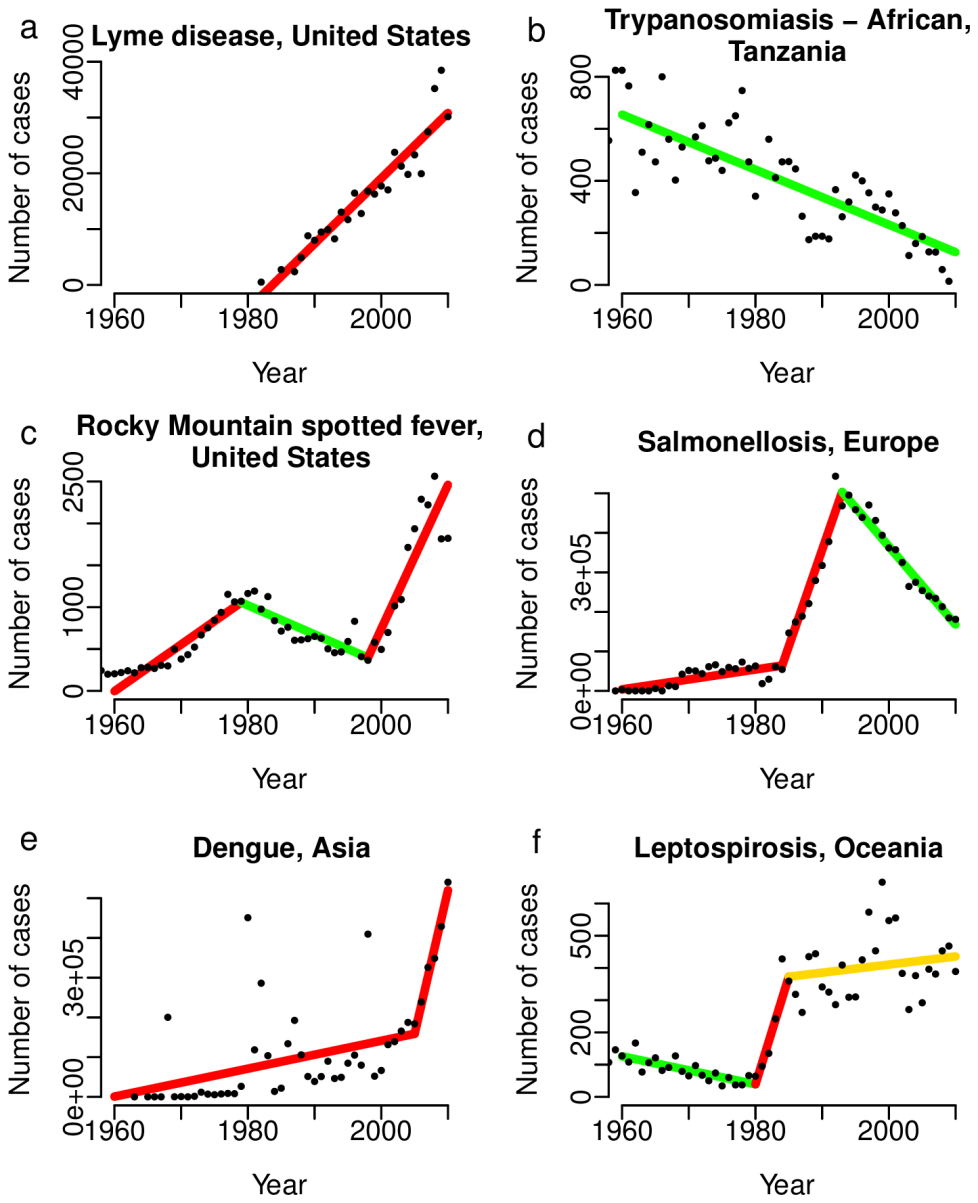
Patterns of incidence illustrating some of the possible outcomes of the proposed analytical framework. A) emergence, b) receding, c) re-emergence, d) receding after emergence, e) emergence and further emergence, f) receding, emergence, stability. Segments in red show significantly positive slopes for that time period, segments in green show significantly negative slopes, and segments in yellow indicate a non-significant trend (p>0.05).

There were six patterns apparent in trends of disease incidence that likely capture most of the variation that is likely to occur. First, incidence trends for several diseases show simple and relatively consistent increases and are characterized as emerging (e.g. Lyme disease in temperate countries over the past three decades; Fig. 1a, S8; Brucellosis in some regions; Fig. S1 in File S1; Rabies in China 1995-present; Fig. S10 in File S1). Second, others show simple monotonic decreases and are clearly receding (African Trypanosomiasis, Tanzania; Fig. 1b). Third, a smaller subset of diseases show an initial significant increase in incidence, a decrease, and then a significant increase, which, as noted above, fits our definition of a ‘re-emerging’ disease (Rocky Mountain Spotted Fever in the USA; Fig. 1c, S11). Fourth, case loads for many diseases show a receding incidence following a previous increase (Salmonellosis in Europe; Fig. 1d, S12 in File S1; Hepatitis B in Europe; Fig. S5 in File S1) and therefore should no longer be described as EIDs. Fifth, case loads for some diseases show an initial increase, then a further increase in the slope (Dengue in Asia, Fig. 1e, and elsewhere, Fig. S3 in File S1). These diseases could be considered to have undergone two phases of emergence without an intervening receding period. Finally, for some diseases there are no significant trends in a given population over a time period identified as distinct via segmented regression, given year-to-year variability (Leptospirosis, Oceania, 1985–2010; p>0.05; Fig. 1f ; Crimean-Congo hemorrhagic fever in Bulgaria and Africa, Fig. S2 in File S1; Hantavirus pulmonary syndrome in the USA, Fig. S4 in File S1). For these diseases there is no evidence for current emergence or receding and they can be termed either stable (if fluctuations are small), non-trending (if fluctuations are large) or if the time series is short, data lacking. Although most of the patterns in Figure 1 are evident upon visual inspection (they were selected to be exemplary), many patterns are not as clear and rigorous analysis is needed to determine the significance of a trend (e.g., the non-significant trend in Fig. 1f, the second increase in incidence of Hepatitis C in Brazil, Fig. S6 in File S1)

It is worth noting that for many diseases, there can be strongly opposing trends in different regions, and in some cases, depending on the population considered. For example, Plague is receding in Brazil and the Americas but is emerging in the Democratic Republic of Congo (Fig. S9 in File S1).

The elevation and trend of a fitted relationship also indicate the relative potential impact of EIDs. For example, while Legionellosis (Fig. S7 in File S1) is emerging in both Europe and the Americas (with similar trends or slopes), the case load is twice as high in Europe, and, all else being equal, greater investment to combat this disease in the population where the case load is higher is warranted. This assumes investment is allocated on the basis of the total number cases rather than the fraction of the population affected, which seems preferable. Similarly, the number of Lyme cases in Poland is both higher and is increasing at a greater rate than in the Czech Republic (Fig. S8 in File S1), which suggests a greater urgency for additional efforts to combat this disease in Poland. These examples use trends in two populations for the same disease to make a valid comparison. Comparing different diseases would require a common currency, such as DALYs.

This approach also provides a way to examine the initial time points of emergence. For example, Legionellosis showed a sharp rise in case numbers in several regions between 1995 and 2001 (Fig. S7 in File S1). If changes in case definitions and detectability of cases can be ruled out, underlying factors that led to emergence during this period might be identified. Alternatively, investigating the cause for a significant rise in cases (i.e., events occuring at a joinpoint) might help in identifying a change in reporting that falsely gives the impression of disease emergence.

## Discussion

Our approach provides a simple method to define a disease as emerging, re-emerging, receding or non-trending, and to describe the magnitude of the rate of change. It provides a tool to implement the conceptual ideas behind previously proposed definitions that were too vague to be widely adopted. If this new method is embraced by the scientific and public health community, it should be possible to overcome the subjective definitions of EIDs used previously and regain the utility of designating diseases as emerging or not.

Our results highlight two key issues. First, the designation of a disease as emerging implicitly refers to longer-term trends than an individual outbreak or epidemic. Data on a yearly timescale is most likely appropriate for decisions involving funding for research, and non-emergency control measures. Most previous studies have not defined the time window over which a disease must increase in incidence to be defined as emerging. This has led to confusion over the terms “emerging” and “re-emerging”, and the exclusion of the latter from some analyses [4], [22], [28], [29]. Our analysis identifies the temporal window (timescale) for a trend statistically rather than arbitrarily, and characterizes trends across the whole time series such that a disease can emerge and re-emerge multiple times depending on the time period of interest.

Secondly, by focusing on a specific, defined population (and by inference, in most cases a geographic region), the analysis provides a more general way of defining an EID than previous studies. Following our approach, diseases can emerge within a population that is a subset of a larger but spatially contiguous population (e.g., a racial group, a gender, a behavioral group, etc.), or within a region (in which case a disease is emerging within that region) or in all humans (in which case the disease is globally emerging). This implies that a disease can be classified as an EID in one population at one scale and be stable or receding in another at a different scale [30]. While this is implied in many studies of EIDs, our approach removes contradictions and ambiguities arising from trying to determine whether disease is either emerging, or not, in all regions at any time.

One issue which merits discussion is the emergence of diseases associated with spatial spread to new regions. Our framework for detecting trends in temporal data (time series) does not directly address spatial spreading of diseases, but it can still provide a broad perspective of temporal trends. For example a disease may invade a new region and fade out (Monkeypox in the USA [31]) or it may become endemic, but with no significant increase in incidence after its invasion of the new region is complete (e.g. West Nile virus in North America from 1999–2012 [32]). Thus, while the spatial spread of these two pathogens is consistent with the definition of an EID that includes the spread of a pathogen to a new region, our framework indicates that neither of these diseases has continued to emerge (i.e. increase in incidence or impact) following their initial spread.

Studies which aim to analyze trends in disease emergence, or the factors that cause them to emerge, require identification of the point in time that a disease first emerges within a population or region [4], [21], [22]. Identifying the precise timing of initial emergence events (i.e., the first few cases of spillover of a novel zoonotic pathogen) is difficult because there can be a substantial time lag between the initial infections and detection, especially for novel diseases in rural or poor populations. For example, evidence suggests HIV infections occurred for several decades before being detected [33]. Our approach identifies emergence as the initial significant rise in incidence or a significant increase in impact and provides a simple method for estimating the time when emergence began – the joinpoint of a segmented regression, or the x-intercept of the initial rise in incidence. It thus provides a strategy to more accurately analyze global trends in EIDs.

In the current analysis, we used annual incidence data to determine whether diseases in specified populations were emerging. If time series data of the impact of diseases are available, and are quantified in a consistent way (e.g, DALYs), this approach could be used to analyze diseases that are thought to have emerged due to increased impact, even without changes in incidence. Our approach can be easily applied to diseases affecting populations of plants, wildlife or livestock.

Using a quantitative approach to determine whether a disease is emerging presents some challenges. First, it requires time series data in a common currency to accurately classify a disease as emerging, and to compare the rate or significance of emergence among different diseases. The ideal would be time series of DALYs for each disease. While this is a challenge for analyzing some historical trends, we believe it is a strength, in that it brings rigor to the analysis. Second, surveillance data, like those used in our example analyses, are influenced by changes in reporting, case definitions, diagnostic capabilities, and other aspects that determine the measured case burden. Third, simple measures like case numbers may be a poor measure of disease impact, especially if populations have different resources for treatment (e.g. AIDS in the USA vs. Africa). As a result, more explicit measures of disease burden should be used whenever possible. Fourth, although we have presented a simple approach based on segmented linear regression, nonlinear approaches may be more appropriate for some data (e.g. Fig. S5 in File S1, Hepatitis B Worldwide).

Translating the results of analyses of trends in disease impact into policy requires careful thought. While the elevation and slope of trends in disease impact provide useful information about the potential impact of a disease in the near future, using trends to predict future impact clearly assumes that past trends will continue, and should be interpreted in the context of current control efforts. For example malaria eradication campaigns have been highly successful in several countries, and analyses of case burdens show strongly receding trends [34]. Diversion of funds away from locations where public health resources are effectively suppressing disease transmission is likely to lead to re-emergence. Specifically, reducing control measures before eradication campaigns are complete due to low and declining case loads is counter-productive. Clearly, the effort and resources currently invested in a disease in a specific population, and the impact of changes in resources allocated on case burdens is required to properly interpret a trend in disease impact.

In summary, over the past two decades, there has been a proliferation in the use of the term “emerging infectious disease”, without a simple and repeatable method to assess emergence. We have proposed a simple quantitative framework to designate a disease as emerging, re-emerging, receding or stable/non-trending and to facilitate comparisons among burdens and trends in different regions, populations and among diseases. This approach allows for the identification of time points associated with changes in case burden that can be used to try to determine the causes of disease emergence. We hope that, with increasingly accurate surveillance data, and given the appropriate context, a quantitative approach like the one suggested here could improve prioritization of resources for infectious disease research, surveillance and control. Implementing this more rigorous definition of an EID will require buy-in and enforcement from scientists, policy makers, peer reviewers and journal editors. Implementation faces significant challenges because doing so will often demonstrate that little evidence exists to support a claim that a favored disease is in fact emerging.

## Supporting Information

File S1 **Number of reported cases per year from the Gideon database (** http://www.gideononline.com/ **) for twelve diseases. Figure S1, Brucellosis. Figure S2, Crimean-Congo hemorrhagic fever. Figure S3, Dengue. Figure S4, Hantavirus pulmonary syndrome. Figure S5, Hepatitis B. Figure S6, Hepatitis C. Figure S7, Legionellosis. Figure S8, Lyme disease. Figure S9,Plague. Figure S10, Rabies. Figure S11, Rocky mountain spotted fever. Figure S12, Salmonellosis.** Maps show countries for which the current trend (ending in 2010) of a disease is emerging (red), receding (green), no significant trend (yellow) or not analyzed (white). The segments of the time series are similarly coded. See Methods for additional details.
(PDF)
