# Supplementary material for: Quantifying Trends in Disease Impact to Produce a Consistent and Reproducible Definition of an Emerging Infectious Disease
Source: PLoS One. 2013 Aug 14;8(8):e69951. doi: 10.1371/journal.pone.0069951 (PMC3743838; doi:10.1371/journal.pone.0069951)
Supplement: File S1 — Number of reported cases per year from the Gideon database ( http://www.gideononline.com/ ) for twelve diseases. Figure S1, Brucellosis. Figure S2, Crimean-Congo hemorrhagic fever. Figure S3, Dengue. Figure S4, Hantavirus pulmonary syndrome. Figure S5, Hepatitis B. Figure S6, Hepatitis C. Figure S7, Legionellosis. Figure S8, Lyme disease. Figure S9,Plague. Figure S10, Rabies. Figure S11, Rocky mountain spotted fever. Figure S12, Salmonellosis. Maps show countries for which the current trend (ending in 2010) of a disease is emerging (red), receding (green), no significant trend (yellow) or not analyzed (white). The segments of the time series are similarly coded. See Methods for additional details. (PDF) [file pone.0069951.s001.pdf]

Supplemental Online Figures

Note: For all figures, data on number of reported cases per year have been obtained from the Gideon database (`http://www.gideononline.com/`). Maps show countries for which the current trend (ending in 2010) of a disease is emerging (red), receding (green), no significant trend (yellow) or no cases reported (white). The segments of the time series are colored in the same way. See methods for additional details.

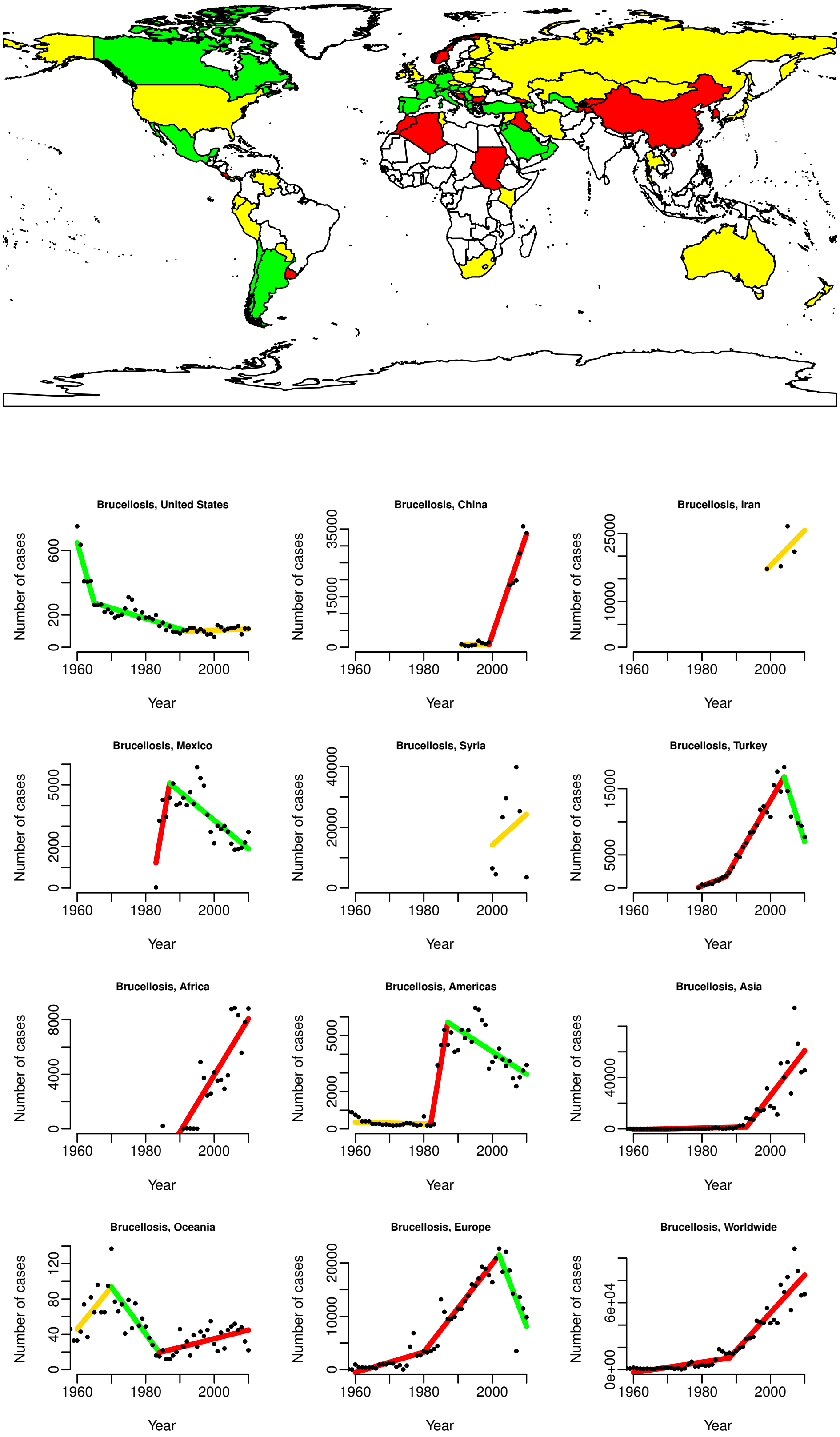

Figure S1: Brucellosis

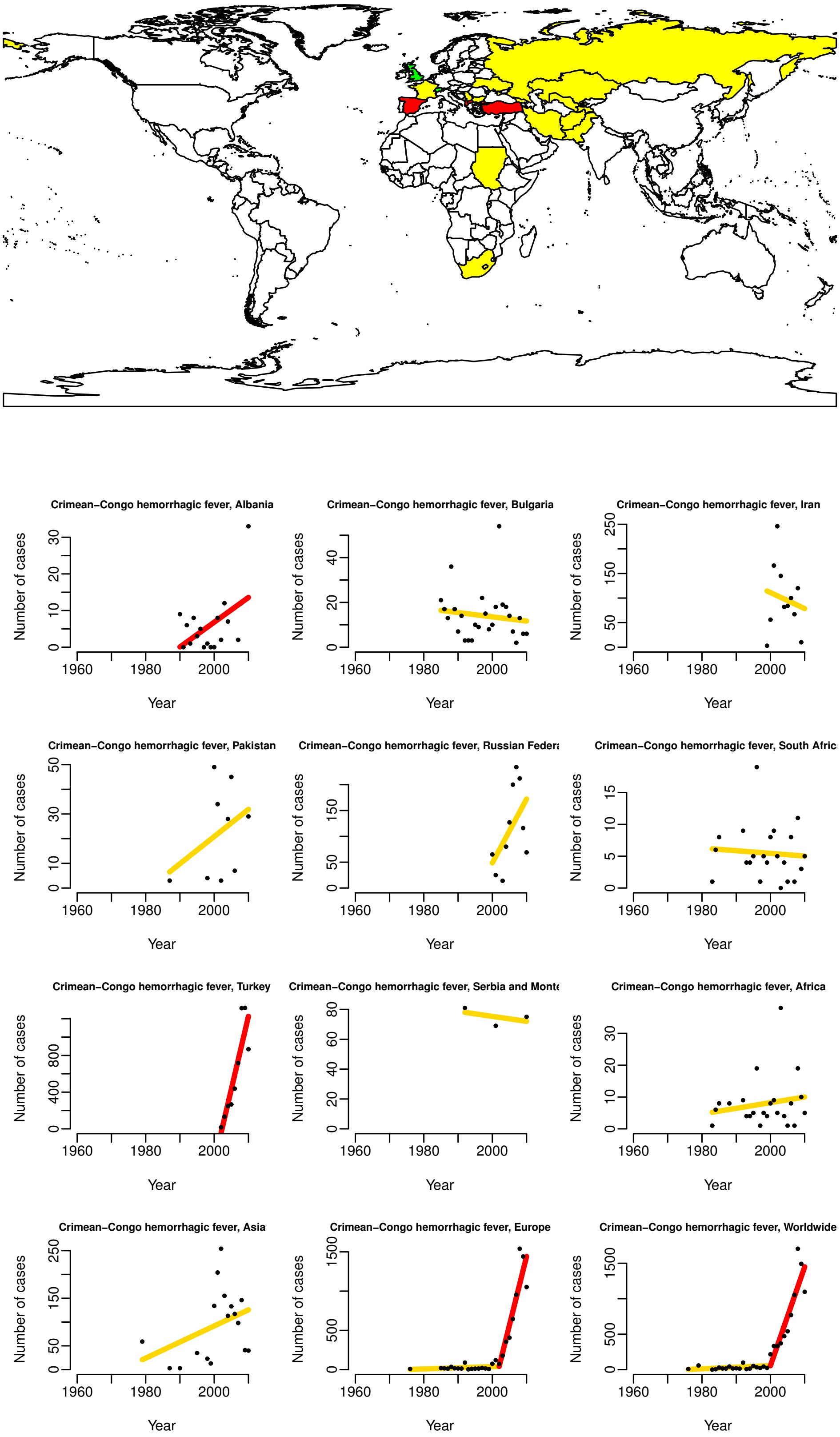

Figure S2: Crimean-Congo hemorrhagic fever

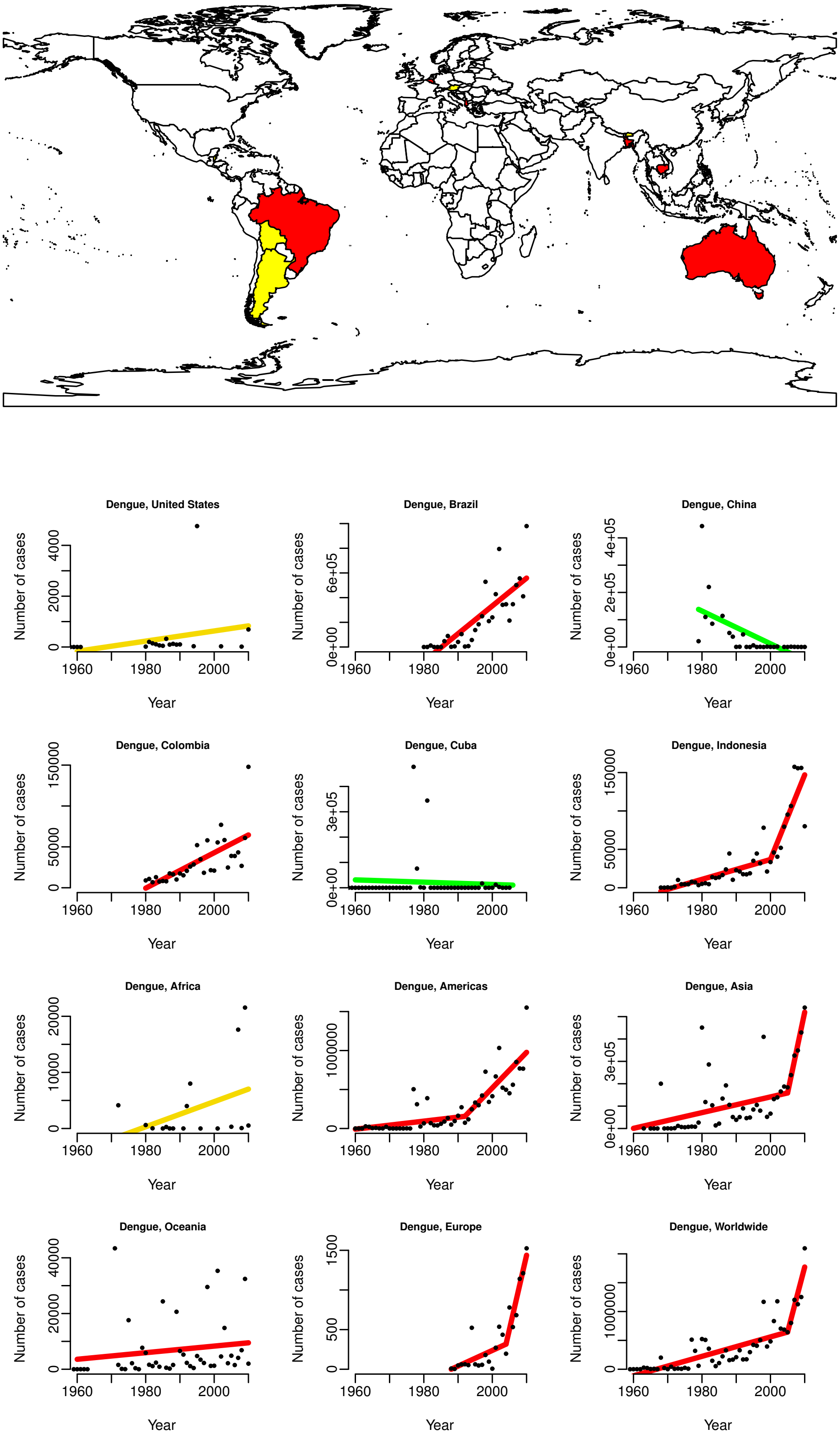

Figure S3: Dengue

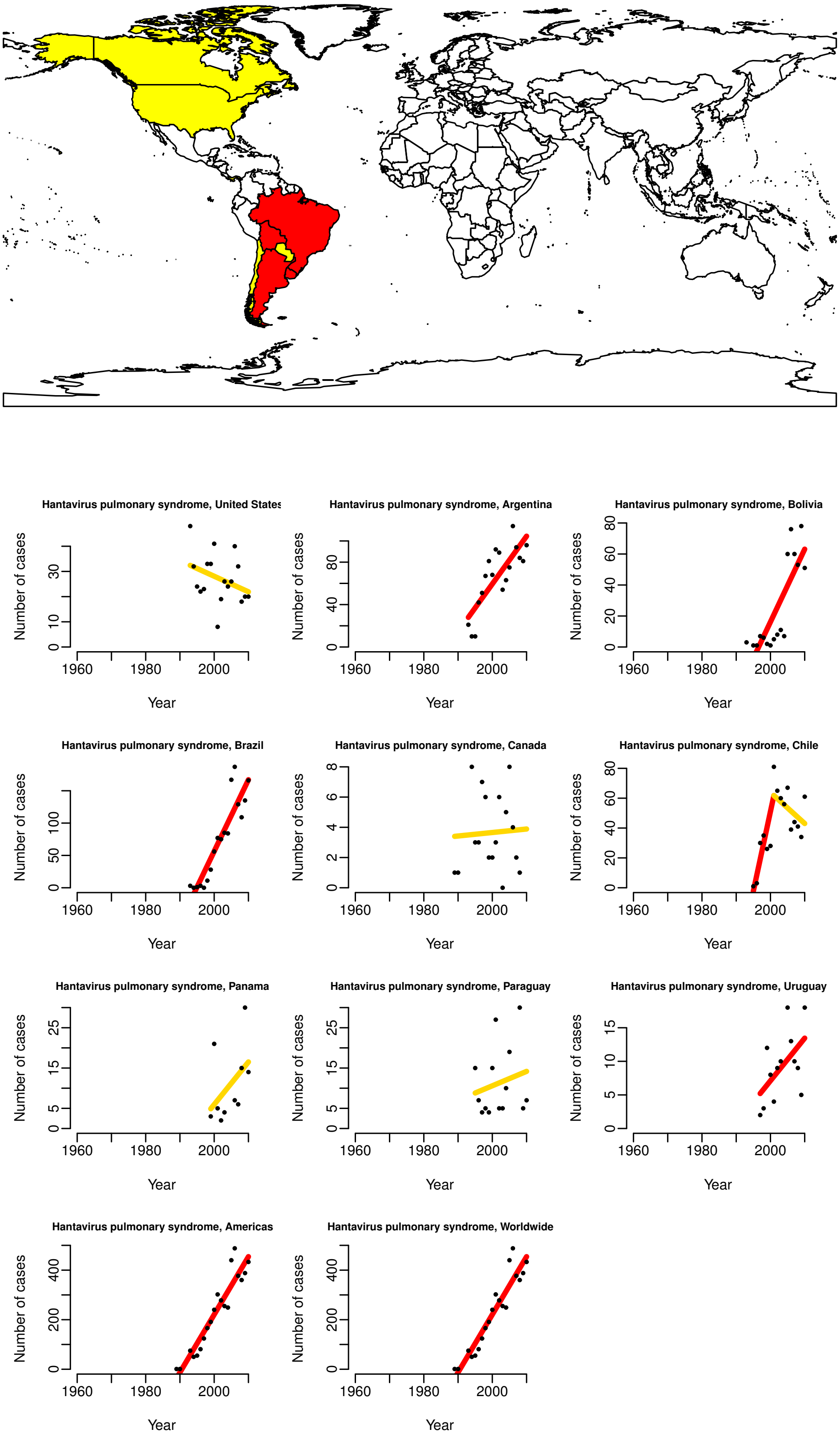

Figure S4: Hantavirus pulmonary syndrome

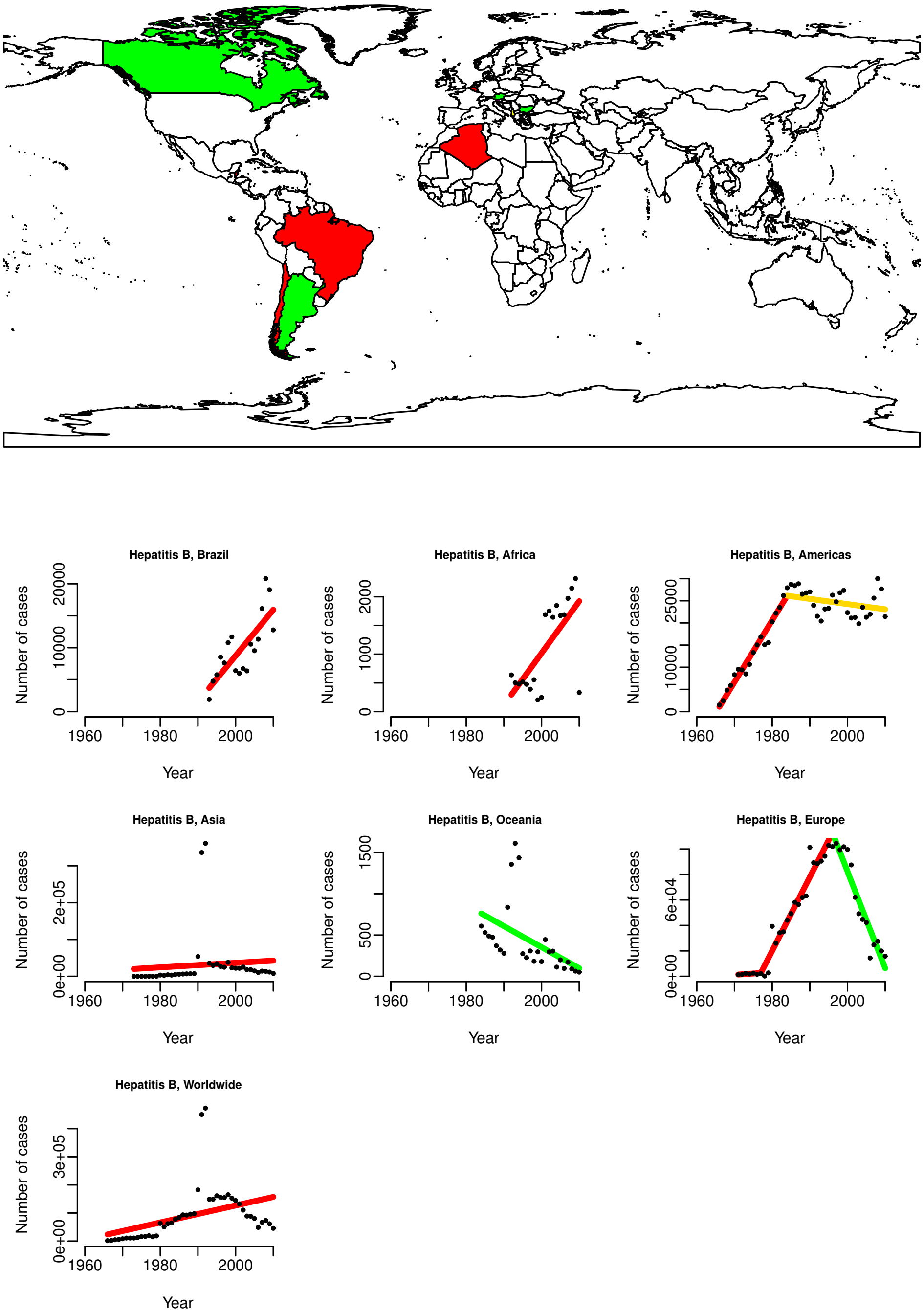

Figure S5: Hepatitis B

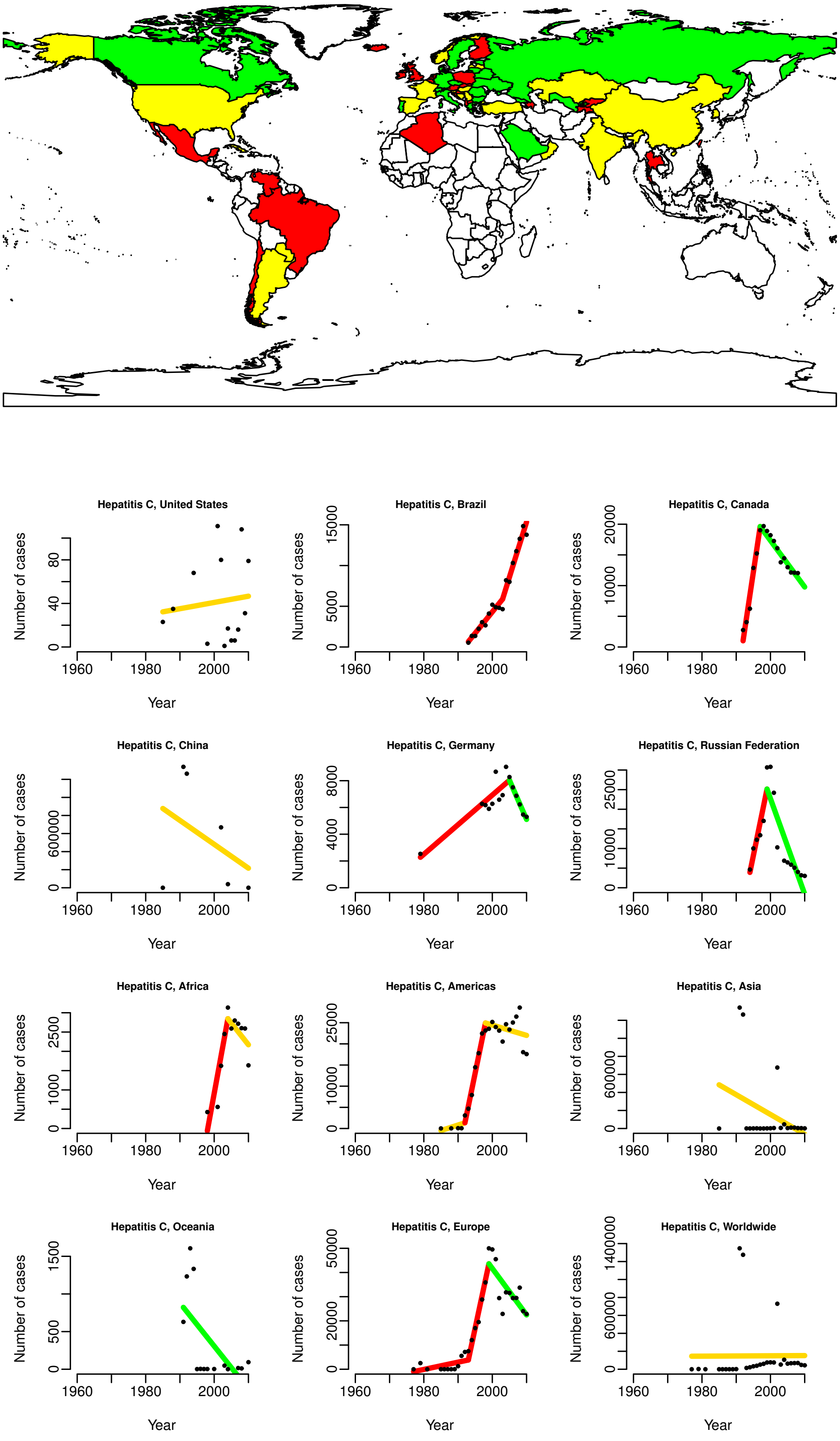

Figure S6: Hepatitis C

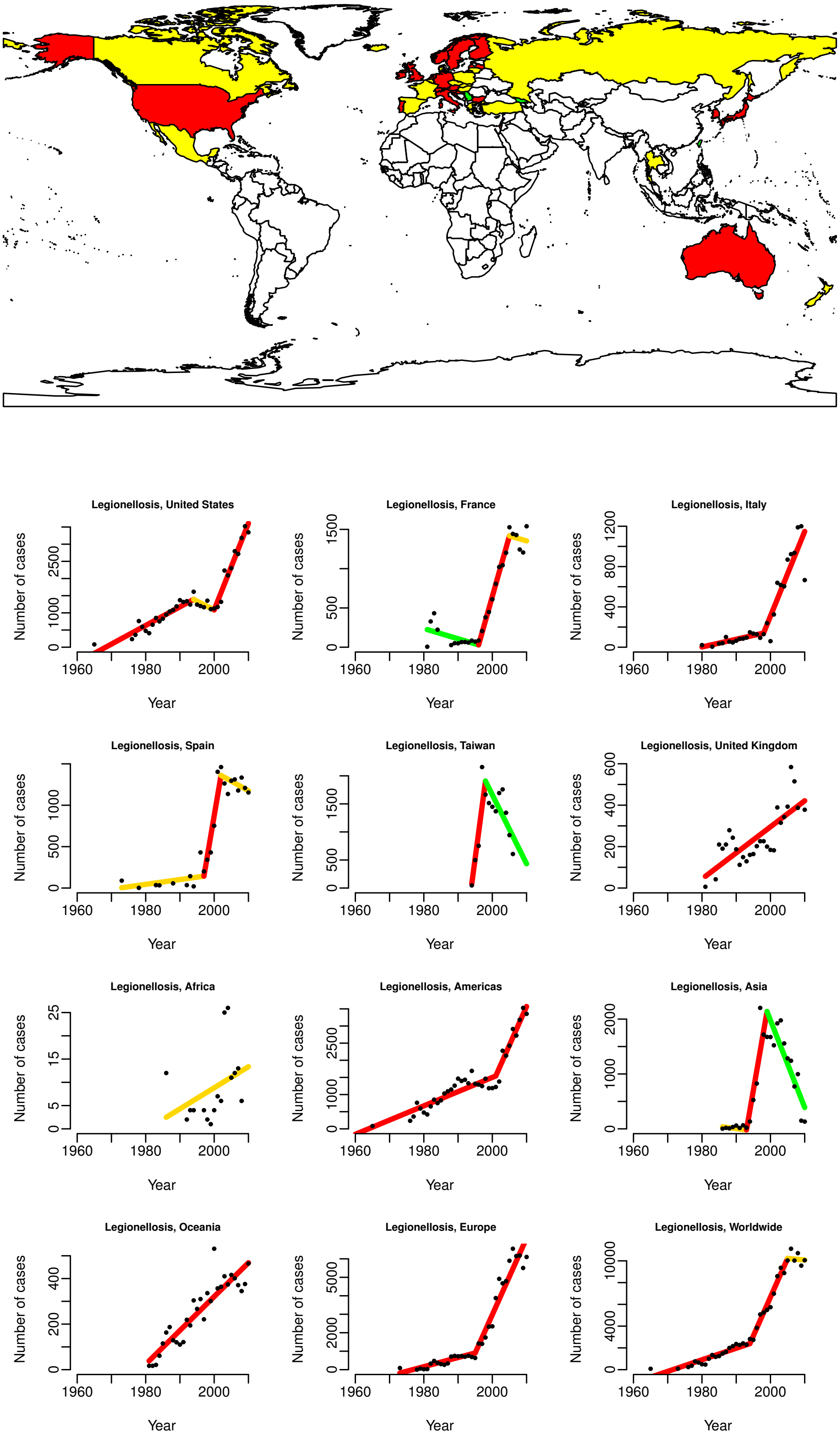

Figure S7: Legionellosis

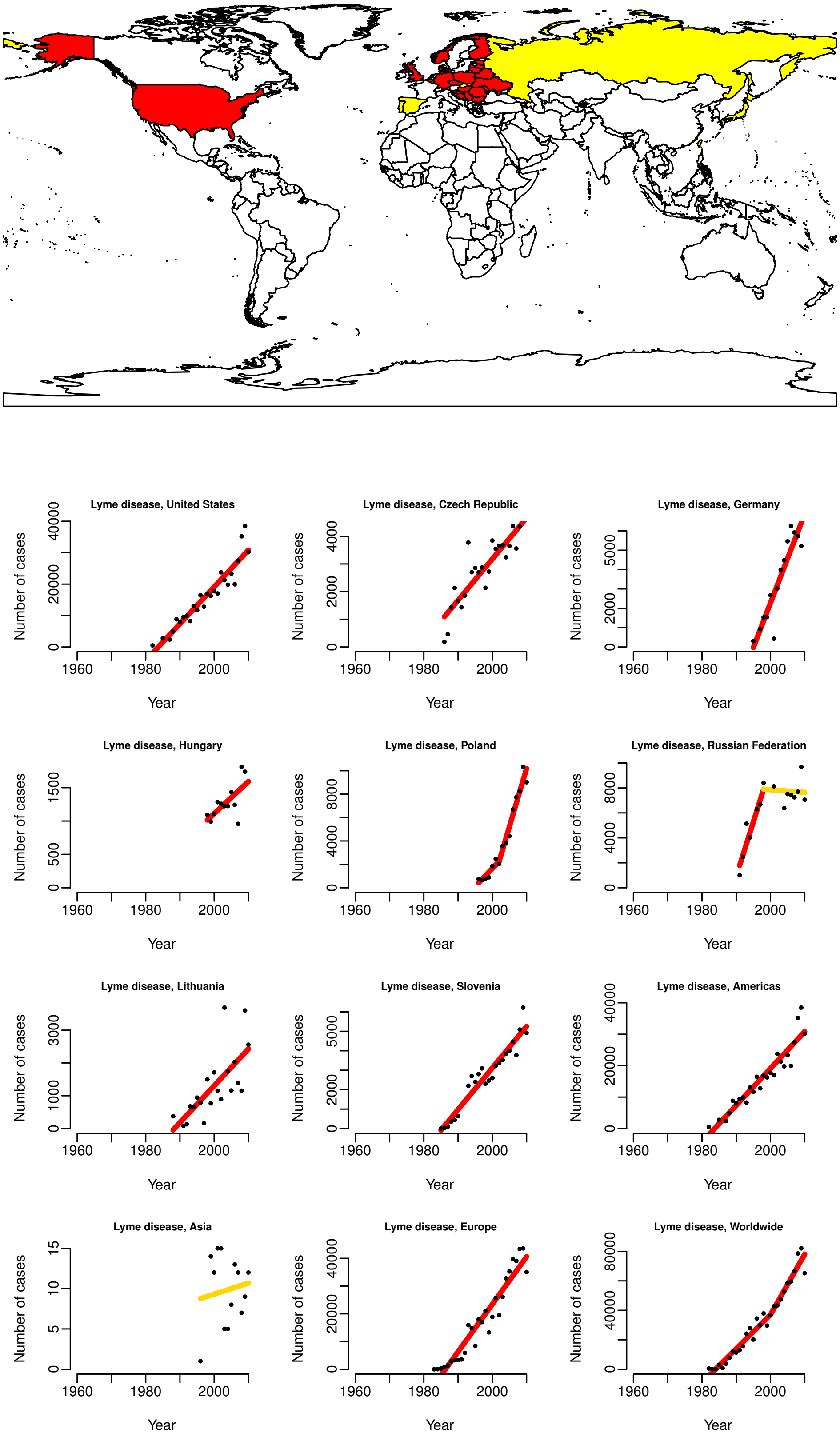

Figure S8: Lyme disease

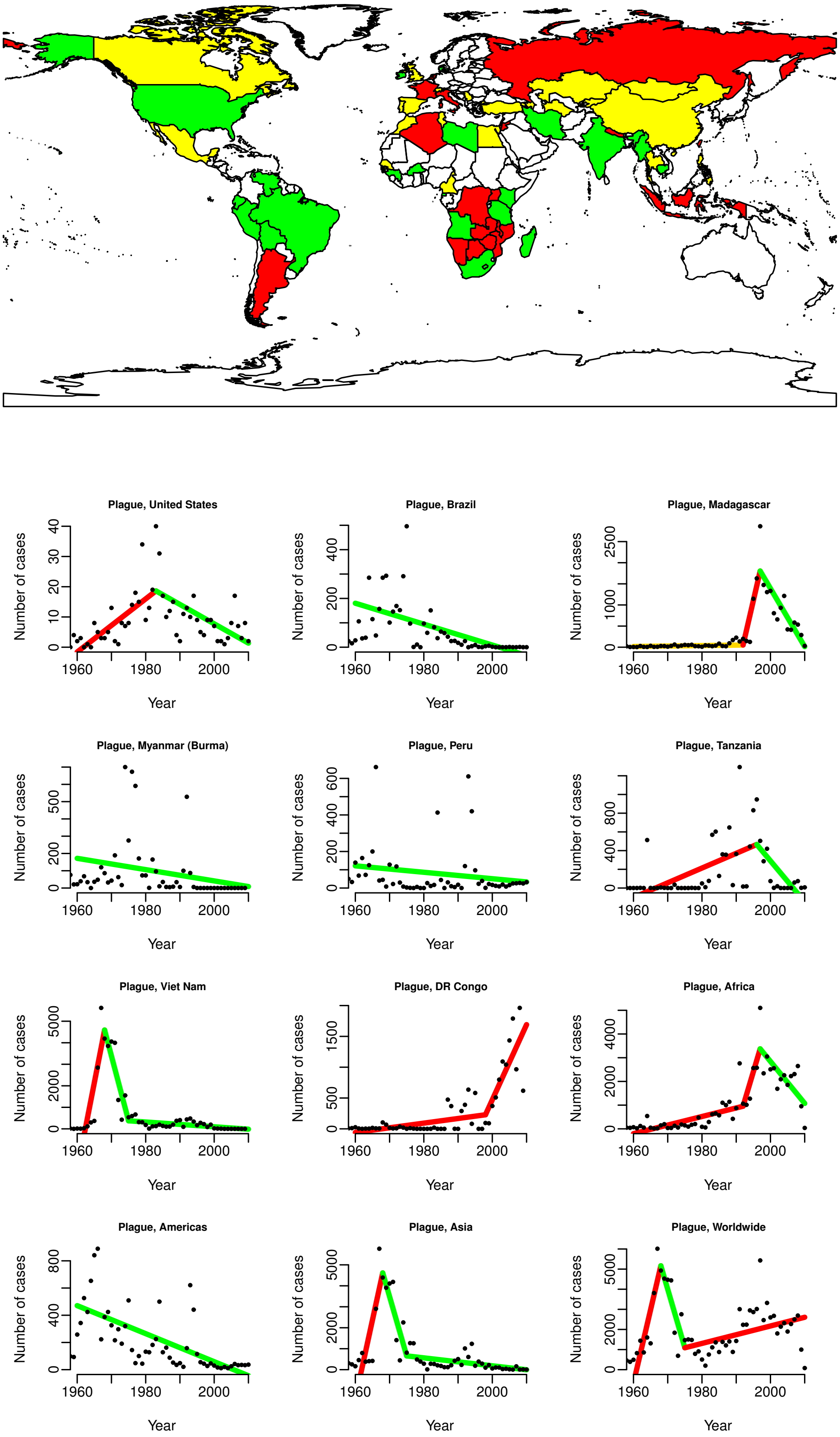

Figure S9: Plague

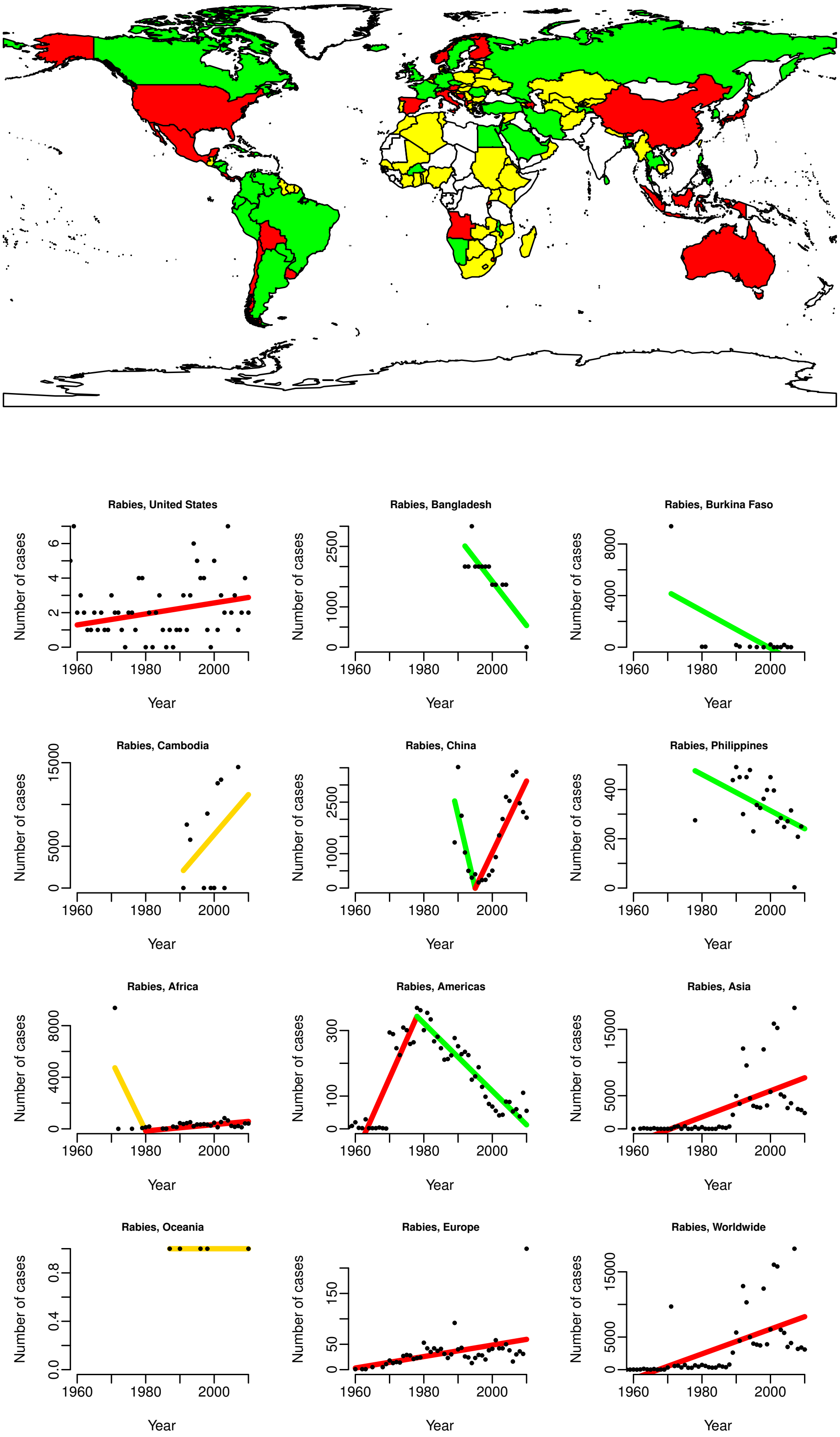

Figure S10: Rabies

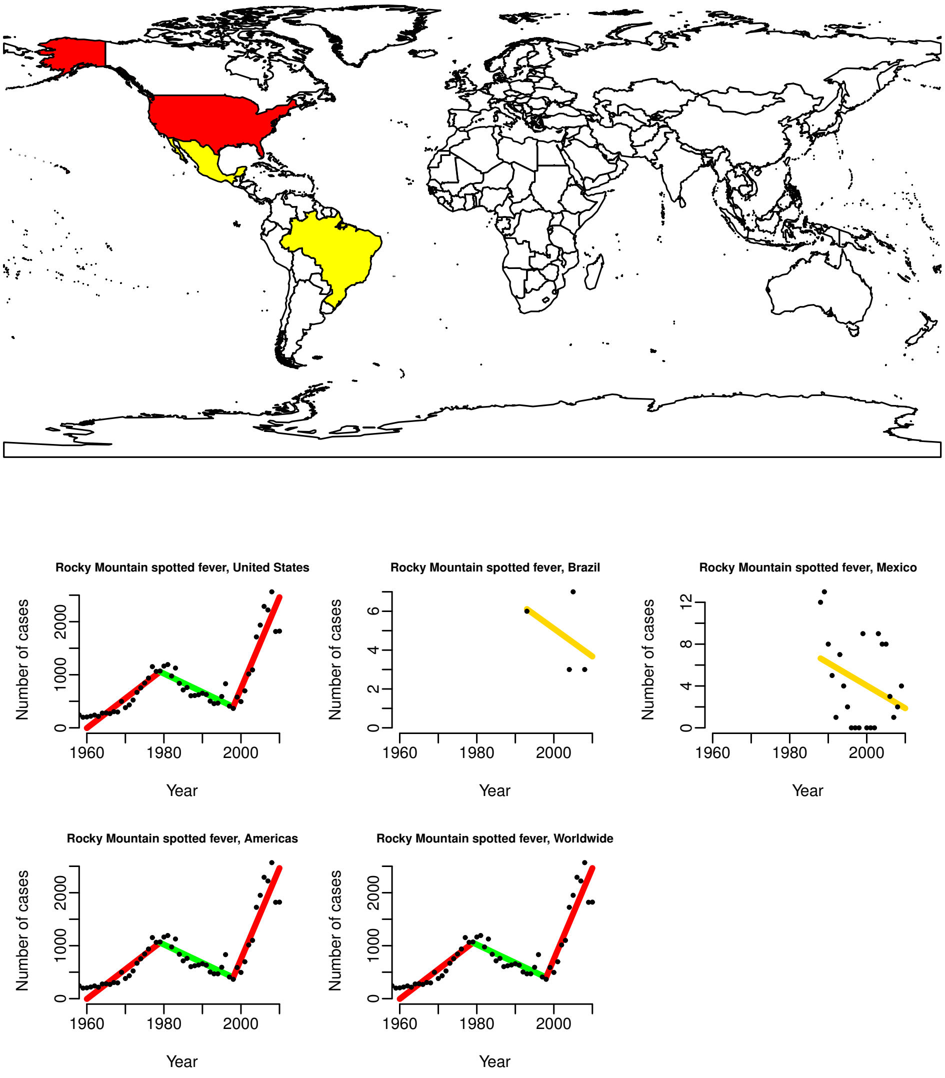

Figure S11: Rocky Mountain spotted fever

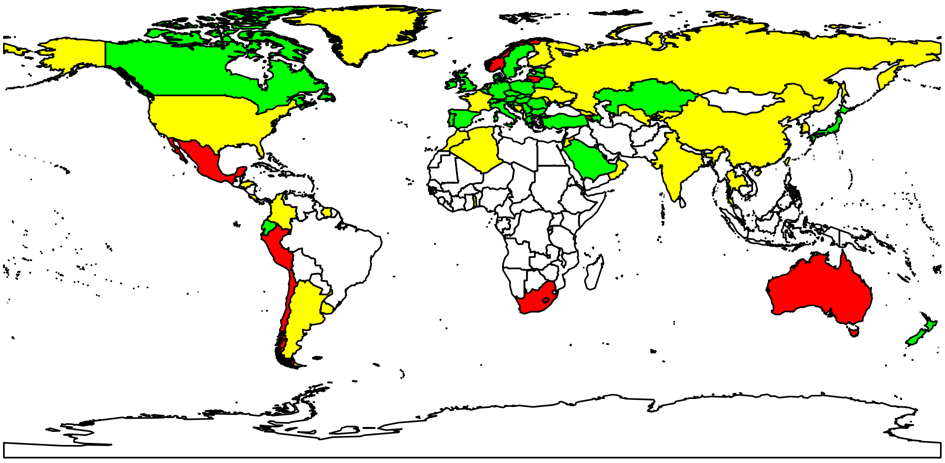

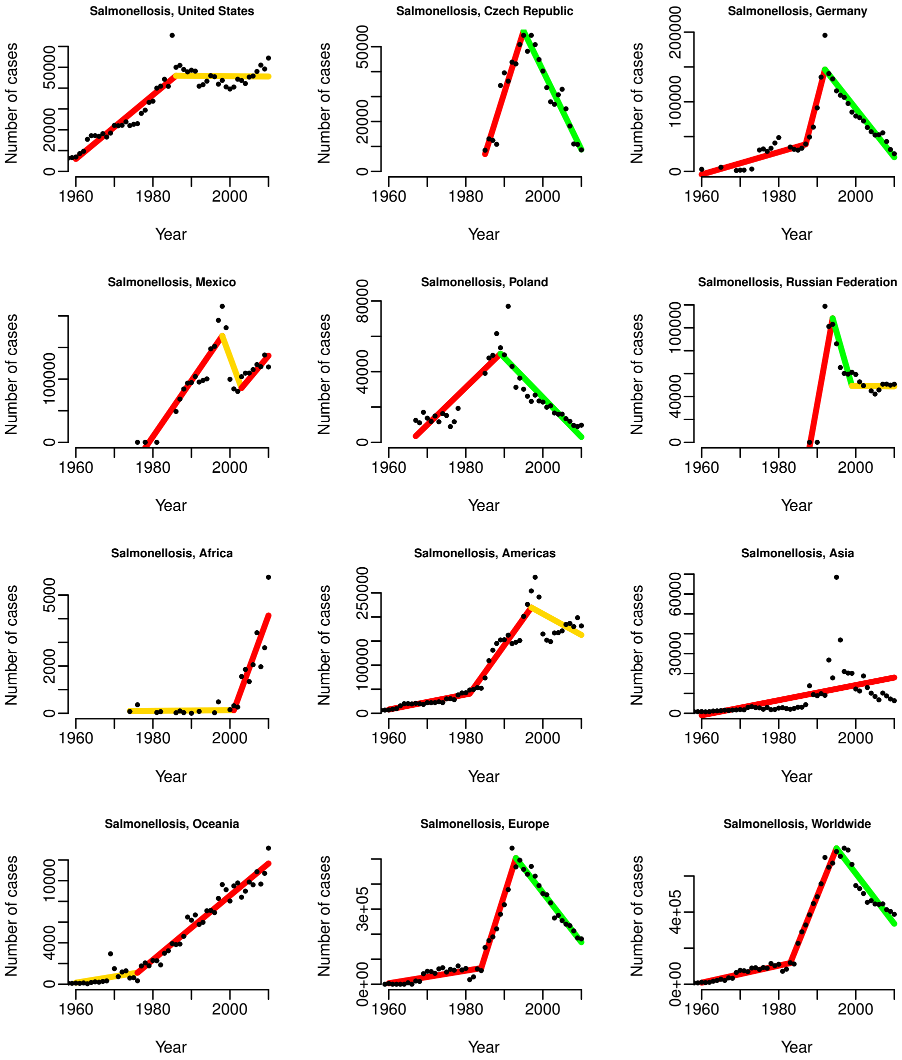

Figure S12: Salmonellosis
